# Genetics and immunity of *Anopheles* response to the entomopathogenic fungus *Metarhizium anisopliae* overlap with immunity to *Plasmodium*

**DOI:** 10.1038/s41598-022-10190-3

**Published:** 2022-04-15

**Authors:** Tullu Bukhari, Vishukumar Aimanianda, Emmanuel Bischoff, Emma Brito-Fravallo, Karin Eiglmeier, Michelle M. Riehle, Kenneth D. Vernick, Christian Mitri

**Affiliations:** 1 Human Health, International Center of Insect Physiology and Ecology (ICIPE), Nairobi, Kenya; 2 Institut Pasteur, Université de Paris, CNRS UMR2000, Molecular Mycology Unit, Department of Parasites and Insect Vectors, F-75015 Paris, France; 3 Institut Pasteur, Université de Paris, CNRS UMR2000, Genetics and Genomics of Insect Vectors Unit, Department of Parasites and Insect Vectors, F-75015 Paris, France; 4 Department of Microbiology and Immunology, Medical College of Wisconsin, Milwaukee, WI 53226 USA

**Keywords:** Genetic association study, Malaria, Innate immunity, Fungal infection

## Abstract

Entomopathogenic fungi have been explored as a potential biopesticide to counteract the insecticide resistance issue in mosquitoes. However, little is known about the possibility that genetic resistance to fungal biopesticides could evolve in mosquito populations. Here, we detected an important genetic component underlying *Anopheles coluzzii* survival after exposure to the entomopathogenic fungus *Metarhizium anisopliae*. A familiality study detected variation for survival among wild mosquito isofemale pedigrees, and genetic mapping identified two loci that significantly influence mosquito survival after fungus exposure. One locus overlaps with a previously reported locus for *Anopheles* susceptibility to the human malaria parasite *Plasmodium falciparum*. Candidate gene studies revealed that two LRR proteins encoded by APL1C and LRIM1 genes in this newly mapped locus are required for protection of female *A. coluzzii* from *M. anisopliae*, as is the complement-like factor Tep1. These results indicate that natural *Anopheles* populations already segregate frequent genetic variation for differential mosquito survival after fungal challenge and suggest a similarity in *Anopheles* protective responses against fungus and *Plasmodium*. However, this immune similarity raises the possibility that fungus-resistant mosquitoes could also display enhanced resistance to *Plasmodium*, suggesting an advantage of selecting for fungus resistance in vector populations to promote naturally diminished malaria vector competence.

## Introduction

*Plasmodium* parasites, the causative agent of malaria, are transmitted by mosquito vectors of the genus *Anopheles*, causing an estimated 229 million cases and 409,000 deaths due to malaria, worldwide in 2019. Currently, vector control is a pillar of the prevention strategy for malaria. However, the heavy reliance on insecticides has led to the evolution and spread of physiological resistance in important vector species ^1^, and insecticide resistance is currently one of the main factors hampering malaria control efforts ^2^. This has directed attention towards development of new vector control tools and their integrated implementation for a sustainable malaria control program ^3,4^.

The idea to complement insecticides with entomopathogenic fungi, such as *Metarhizium anisopliae* and *Beauveria bassiana,* has gained attention largely due to the fact that the evolutionary pressure to evolve resistance may be lower than for chemical insecticides ^5,6^. Entomopathogenic fungi tend to kill mosquitoes after they reproduce and thus selection pressure is weaker than for rapidly killing chemical insecticides ^7–11^. Entomopathogenic fungi have also been shown to be effective against insecticide-resistant mosquito species ^8^. In addition technical progress in fungus production, formulation and delivery to mosquitoes are promising ^12–14^. Entomopathogenic fungi, unlike other microbes such as bacteria and viruses, do not need to be ingested by the host. Fungus spores infect by contact with the insect cuticle, producing a germ tube to invade and grow in the insect hemocoel by secreting cuticle-degrading enzymes followed by growth and release of toxins ^10,11^.

In insects, cellular and humoral antifungal immune responses have been described, but whether genetic variation in insect populations could shape the insect antifungal response is not well known. Mosquito genetic variation significantly affects the outcome of infection with *Plasmodium* parasites ^15^. In nature, mosquitoes are routinely exposed to a variety of fungi, and mosquito genetic variation could plausibly contribute to differential fungus susceptibility. While entomopathogenic fungi are evolutionarily distant from *Plasmodium*, aspects of mosquito immunity to both pathogens may be shared, as both are eukaryotes ^16^.

Here we performed a familiality study to determine if genetic factors influence mosquito survival after infection with entomopathogenic fungus, followed by a genetic mapping study to identify genetic loci contributing to the differential survival revealed by the familiality study. In a functional dissection of the most significant mapped locus, we explore the role of candidate genes and interacting functional partners as antifungal factors. We found that leucine-rich repeat (LRR) coding proteins and their interacting partners TEP1, TEP3 displayed a protective function against the entomopathogenic fungus *M. anisopliae*.

## Results

### Genetic basis for differential mosquito survival of fungus infection

We investigated the genetic dependence of mosquito mortality after fungus infection in two stages. First, in a familiality study, we compared rates of mortality between mosquito isofemale pedigrees that were each generated by a different founding pair of wild mosquitoes. The goal was to measure inter-pedigree differences for the phenotype of fungus mortality in pedigrees exposed simultaneously to fungus under the same environmental conditions.

Six isofemale pedigrees of *A. coluzzii* collected in Burkina Faso were simultaneously challenged with the same dose of *M. anisopliae* ICIPE30. If there were no segregating mosquito genes that influence the fungus infection mortality phenotype, then mortality curves of the different pedigrees should display similar distributions. Conversely, the action of genes that influence fungus infection mortality should cause significant pedigree-specific changes in the mortality curves. Replicate infections in the same mosquito line using the same dose of fungus under controlled environmental conditions generated statistically indistinguishable mosquito mortality curves ^17,18^, indicating low levels of experimental noise in the assay system and supporting the interpretation that significantly different curves among pedigrees would be strongly suggestive of a genetic basis for phenotypic differences.

**Figure 1:**
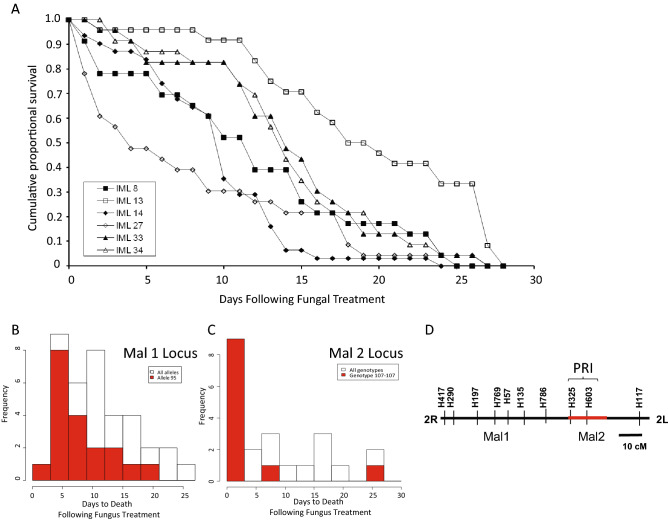
Genetic basis for differential mosquito survival following fungus infection. (**A**)**.** Mosquito mortality curves for six age-matched isofemale pedigrees challenged with the same dose of *M. anisopliae* were significantly different, with median time to death from 4 to 20 days (p < 0.001, Kaplan Meier Survival Analysis and Log-Rank non-parametric comparison). (**B**). Mapping of locus *M. anisopliae* longevity (Mal) 1 displayed as histogram of fungal mortality phenotype of *A. coluzzii* pedigree JBC03 (origin, Mali) by microsatellite marker 2R.H769 alleles. Red bars show phenotypic distribution for the carriers of allele 95 at 2R.H769. Open bars show the overall phenotypic distribution for all phenotypes of the family combined. Individuals carrying allele 95 (red bar) displayed average time to death = 8.4 d, which was significantly faster (genome-wide adjusted p-value = 0.05) than individuals carrying alternate alleles (open bars; allele 88 = 11.7 d to death, allele 109 = 13.0 d to death). (**C**). Mapping of locus Mal2 displayed as histogram of fungal mortality phenotype of *A. coluzzii* pedigree IML27 (origin, Burkina Faso) by microsatellite marker 2L.H603 genotypes. Individuals carrying genotype 107–107 (red bar) displayed average time to death = 4.6 d, which was significantly faster (genome-wide adjusted p-value = 0.02) than individuals carrying any other genotype (open bars; alternate genotypes = 13.8 d to death). (**D**). Chromosome 2 linkage map of *A. coluzzii* indicating genomic positions of loci linked with significant effect on survival following fungal infection in wild isofemale pedigrees from West Africa. Mal1 (*M. anisopliae* longevity 1) is on chromosome arm 2R while Mal2 is on arm 2L. Horizontal line indicates arms of chromosome 2 (R, right arm; L, left arm), and vertical lines indicate microsatellite markers. Red line indicates location of the major genomic locus controlling the outcome of infection with *P. falciparum* indicated by PRI with bracket for Plasmodium Resistance Island ^15^. Scale bar, 10 centiMorgans.

Mosquito mortality was significantly different across the fungus-exposed *A. coluzzii* pedigrees, with median time to death ranging from 4 to 20 days (p < 0.001, Kaplan Meier Survival Analysis and Log-Rank non-parametric comparison; Fig. 1A). Thus, the familiality study provided strong evidence for a genetic component influencing mosquito survival of infection with *M. anisopliae*. Because the pedigrees were initiated from wild-collected mosquitoes, the result indicates that the fungus survival phenotype is influenced by alleles segregating in the natural population of *A. coluzzii*, and that such alleles are collectively frequent in nature. This information was not previously known for *Anopheles*, and led to more detailed examination in the second stage of this study.

In the second stage, we performed a genome-wide genetic mapping scan for loci that influence the survival response of mosquitoes after *M. anisopliae* exposure. The six *A. coluzzii* pedigrees from Burkina Faso shown in Fig. 1A and one pedigree from Mali, *A. coluzzii* JBC03, were analyzed. For each individual mosquito, the fungus response phenotype was the number of days after fungus exposure until death, and the genotype was determined using a genome-wide marker map.

Statistical comparison of the survival phenotype distribution with the genome-wide genotypes detected two significant loci with explanatory power for time of mosquito survival following fungal infection. In *A. coluzzii* pedigree JBC03, individuals carrying allele 95 of microsatellite locus H769 on chromosome 2R displayed an average time to death of 8.4 d, which was significantly faster than individuals carrying alternate alleles at H769 that died in 11.7 d for allele 88 or 13.0 d for allele 109 (genome-wide adjusted p-value = 0.05; Fig. 1B). This locus was named *M. anisopliae* longevity (Mal) 1. In *A. coluzzii* pedigree IML27, individuals carrying the genotype 107–107 at microsatellite locus H603 on chromosome 2L displayed an average time to death of 4.6 d, which was three times faster than mosquitoes carrying any other genotype at H603 that died in an average of 13.8 d (genome-wide adjusted p-value = 0.02; Fig. 1C). This locus was named Mal2. Significant loci were not detected in the other five *A. coluzzii* pedigrees (adjusted p-value for IML8, 0.7; IML13, 0.1; IML14, 0.3, IML33, 0.2. IML34, 0.7). The absence of significant linkage detected in these pedigrees could mean that the genetic influence upon fungal survival in these pedigrees is multigenic or complex, or that an informative mapping marker was not close enough to a putative causative locus. Further work would be required to genotype these pedigrees with a denser marker map in order to clarify the underlying genetics.

Both loci mapped are located on chromosome 2, with Mal1 on the right arm and Mal2 on the left arm (Fig. 1D). Interestingly, the Mal2 locus partially overlaps a previously mapped locus with a significant effect on *Plasmodium falciparum* infection outcome in the natural populations of *A. coluzzii* and *A. gambiae* in West and East Africa, named the Plasmodium Resistance Island or PRI ^15,19^. The Mal2 locus displays a stronger genetic effect than Mal1 for fungus infection survival in terms of the p-value of the locus, and Mal2 genotypes also display a high explanatory power for the fungus survival phenotype because presence of the susceptible allele 107 or genotype 107–107 at the Mal2 locus was sufficient to explain ≥ 87% of mosquitoes dying in the first 5 days post-infection. Based on the stronger effect of Mal2 on fungus infection survival, and because of its physical proximity to the PRI locus for malaria susceptibility, the Mal2 locus on chromosome 2L was prioritized for further functional examination by candidate gene studies.

### Mal2 locus LRR-coding genes and functional partners influence fungus infection outcome

**Figure 2:**
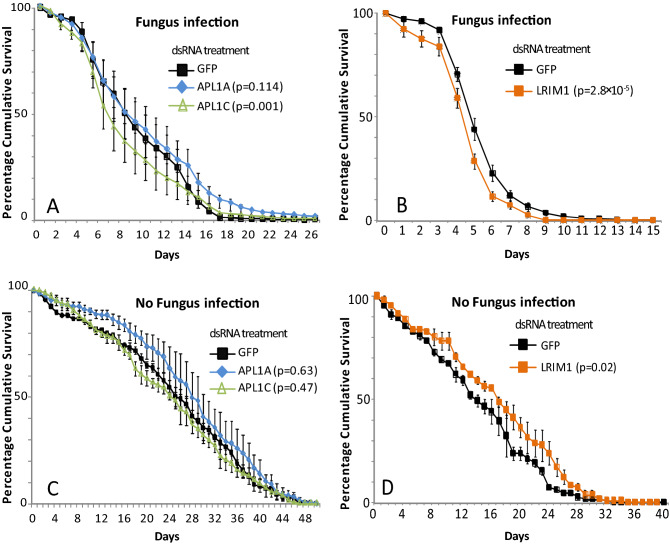
Leucine-rich repeat protein genes in the Mal2 locus influence fungus infection outcome. Graphs illustrate mortality curves of female mosquitoes after gene silencing followed or not with fungal infection. (**A**) Silencing of APL1A and APL1C gene expressions followed by infection with *M. anisopliae,* (**B**) Silencing of LRIM1 genes expression followed by infection with *M. anisopliae,* (**C**) Silencing of APL1A and APL1C gene expressions without fungal infection (**D**) Silencing of LRIM1 genes without fungal infection. P-values obtained from Cox Regression testing for difference between the given mortality curve and the dsGFP mortality curve are given in the legend. The y-axis coordinates on each panel represent the range of lifespan for the given condition. Error bars represent the variability between three independent biological replicates. For each tested gene, 50 mosquitoes were tested in each of three biological replicates.

Candidate genes of known immune interest are located in physical proximity to the Mal2 locus, in particular the leucine-rich repeat protein (LRR) encoding genes APL1A, APL1C and LRIM1, which mediate potent host protection against malaria parasite infection ^15,20–22^. Here, we tested phenotypes of these LRR protein genes for host protection from fungal infection by silencing gene expression using treatment with specific or control double-stranded RNA (dsRNA), challenging mosquitoes with fungal exposure, and measuring mortality as compared to controls. Depletion of APL1C by treatment of mosquitoes with dsAPL1C decreased mosquito survival after infection with *M. anisopliae*, indicating that activity of APL1C is required for protection against fungal infection (p = 0.001, Cox HR = 1.25, 99% CI 1.05–1.5; Table 1 and Fig. 2A). Similarly, silencing of the LRR protein gene LRIM1 decreased mosquito survival after infection with *M. anisopliae* (p = 2.8 × 10^–5^, Cox HR = 1.3, 99% CI 1.1–1.5; Table 1 and Fig. 2B). In contrast, depletion of LRR protein APL1A had no effect on survival (p = 0.114, Cox HR = 0.9, 99% CI 0.75–1.1; Table 1 and Fig. 2A). As a control, we tested dsRNA treatments in the absence of fungal infection, and mosquito survival was not different between dsGFP and dsAPL1C treatments (p = 0.47, Cox HR = 1.1, 99% CI 0.8–1.4; Table 1 and Fig. 2C), dsGFP and dsAPL1A treatments (p = 0.63, Cox HR = 0.8, 99% CI 0.6–1.1; Table 1 and Fig. 2C), or dsGFP and dsLRIM1 treatments (*p* = 0.02, Cox HR = 0.7, 99% CI 0.5–1.0; Table 1 and Fig. 2D). These results indicate that the immune LRR proteins APL1C and LRIM1 but not APL1A are required for mosquito protection from mortality due to infection with *M. anisopliae*.

**Table 1:** Hazard ratio HR (99%CI) and lethal median time (LT50) of adult female *A. coluzzi* mosquitoes after gene silencing by dsRNA treatment.

dsRNA	Fungus Infection					dsRNA	No Fungus Infection				
	HR	99% CI	*p*	LT50 ± S.E	n		HR	99% CI	*p*	LT50 ± S.E	n
Cactus	0.65	0.5–0.9	**1.2 × 10**^**–4**^	4 ± 0.14	150	Cactus	1.6	1.2–2.1	**1.5 × 10**^**–6**^	9 ± 0.5	150
Rel2-RHD	1.1	0.8–1.4	0.395	6 ± 0.24	164	Rel2-RHD	1.1	0.9–1.4	0.23	11 ± 0.5	211
Rel2-ANK	1.6	1.3–2.0	**2.9 × 10**^**–8**^	4 ± 0.09	292	Rel2-ANK	1.1	0.9–1.4	0.28	12 ± 0.5	355
APL1A	0.9	0.7–1.1	0.114	8 ± 0.34	445	APL1A	0.8	0.6–1.1	0.63	28 ± 0.7	218
APL1C	1.2	1.05–1.5	**0.001**	7 ± 0.28	433	APL1C	1.1	0.8–1.4	0.47	25 ± 1.3	182
LRIM1	1.3	1.1–1.5	**2.8 × 10**^**–5**^	5 ± 0.71	427	LRIM1	0.7	0.5–1.0	0.02	17 ± 1.0	110
TEP1	1.3	1.03–1.5	**0.003**	5 ± 0.10	191	TEP1	1.3	1.0–1.6	0.01	13 ± 0.5	207
TEP3	0.9	0.7–1.01	0.19	5 ± 0.12	459	TEP3	1.1	0.8–1.5	0.34	26 ± 0.6	127
TEP4	1.1	0.9–1.3	0.05	5 ± 0.03	418	TEP4	1.1	0.8–1.5	0.22	26 ± 0.3	182

*P*-values (*p*) were obtained from the Cox regression model. n corresponds to the total number of mosquitoes used for the Kaplan–Meier.

Significant values are in bold.

**Figure 3:**
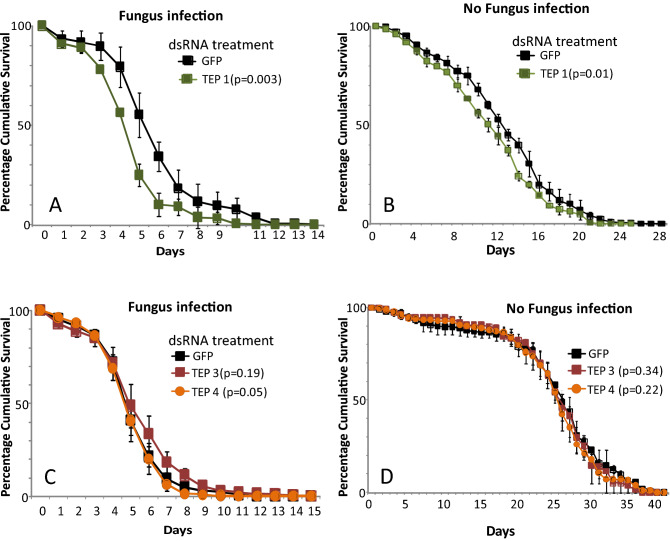
Functional partners of Mal2 locus LRR genes also participate in antifungal protection. Graphs illustrate mortality curves of female mosquitoes after gene silencing followed or not with fungal infection. (**A**) Silencing of Tep1 gene expression followed by infection with *M. anisopliae* (**B**) Silencing of Tep1 gene expression without fungal infection (**C**) Silencing of TEP3 and Tep4 gene expression followed by *M. anisopliae* infection (**D**) Silencing of TEP3 and Tep4 gene expression without fungal infection. P-values obtained from Cox Regression are given in brackets in the legend. Error bars represent the variability between three independent biological replicates. For each tested gene, 50 mosquitoes were tested in each of three biological replicates.

The LRR proteins APL1C and LRIM1 are known to form a ternary complex with the complement-like protein TEP1, and activity of all three components of the complex are required for killing of malaria parasites in the mosquito midgut ^23,24^. Because we found that APL1C and LRIM1 are also required for full protection from fungus-dependent mortality, we tested whether the TEP1 complex partner required for killing of malaria parasites is also involved in antifungal protection. Silencing of TEP1 expression produced the same phenotype of increased mosquito mortality after infection with *M. anisopliae* (p = 0.003, Cox HR = 1.3, 99% CI 1.03–1.5; Table 1 and Fig. 3A). In the control case, mosquito survival was not different between dsGFP and dsTEP1 (p = 0.01, Cox HR = 1.3, 99% CI 1.0–1.6; Table 1 and Fig. 3B). This result indicates that the complement-like system based on TEP1 as well as the two LRR proteins APL1C and LRIM1 protects not only against *Plasmodium* but also against fungal infection.

It was previously shown that the LRR protein complex comprised of APL1C and LRIM1 can also physically interact with other TEP family proteins such as TEP3 and TEP4 in order to protect against infection with rodent or human malaria parasite species, respectively ^23,25^. However, neither protein displayed strong effect on fungus-dependent mortality in female *Anopheles* (TEP3, p = 0.19, Cox HR = 0.9, 99% CI 0.7–1.01; Table 1 and Fig. 3C; TEP4, p = 0.05, Cox HR = 1.1, 99% CI 0.9–1.3; Table 1 and Fig. 3C). In control mosquitoes without fungus exposure, mosquito survival was not different between the control dsGFP and dsTEP3 (p = 0.34, Cox HR = 1.1, 99% CI 0.8–1.5; Table 1 and Fig. 3D nor between dsGFP and dsTEP4 (p = 0.22, Cox HR = 1.1, 99% CI 0.8–1.5; Table 1 and Fig. 3D).

### Toll and Imd immune pathways protect Anopheles against fungal infection

**Figure 4:**
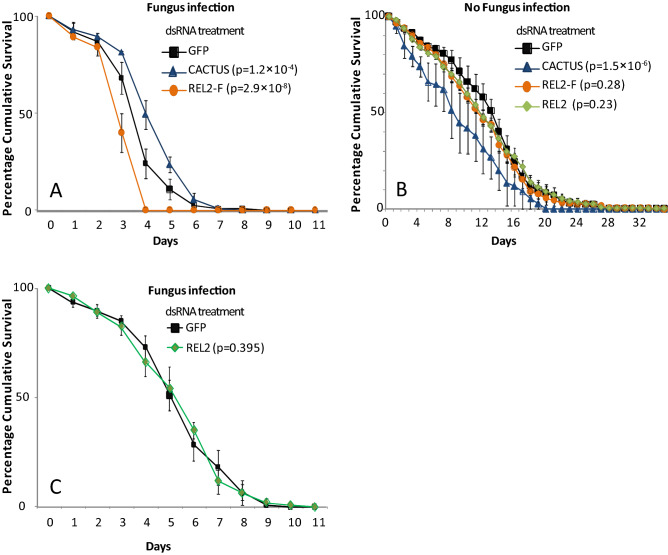
Toll and Imd immune pathways protect *Anopheles* against fungal infection. Graphs illustrate mortality curves of female mosquitoes after gene silencing followed or not with fungal infection. (**A**) Silencing of CACTUS and REL2-F gene expression followed by infection with *M. anisopliae* (**B**) Silencing of CACTUS, REL2-F and REL2 gene expression without fungal infection (**C**) Silencing of REL2 gene expression followed by infection with *M. anisopliae*. P-values obtained from Cox Regression are given in brackets in the legend. Error bars represent the variability between three independent biological replicates. For each tested gene, 50 mosquitoes were tested in each of three biological replicates.

The above-tested APL1 family genes in the Mal2 locus, as well as the functional protein partner complement-like TEP1, are regulated by the Toll and Imd immune signaling pathways ^15,20,22,26^. Therefore, we determined whether these signaling pathways influence mosquito protection against *M. anisopliae*. The Toll pathway was experimentally activated by treatment of mosquitoes with dsRNA targeting its negative regulator, Cactus (dsCactus). Activation of the Toll pathway augmented mosquito survival after exposure to *M. anisopliae* as compared to controls treated with dsGFP (p = 1.2 × 10^–4^, Cox HR = 0.67, 99% CI 0.5–0.9; Table 1 and Fig. 4A). In control mosquitoes without fungus exposure, Toll activation by dsCactus caused lower survival as compared to dsGFP treatment (p = 1.5 × 10^–6^, Cox HR = 1.6, 99% CI 1.2–2.1; Table 1 and Fig. 4B), indicating an inherent fitness cost of Toll activation and therefore a greater effective magnitude of the difference in survival observed in Toll-activated mosquitoes after fungus exposure.

The *Anopheles* Imd pathway is regulated by the Nuclear factor kappa B (NF-κB) transcription factor Rel2, which is expressed as a full-length isoform (Rel2-F) and a short isoform (Rel2-S). Gene silencing of Rel2-F alone decreased mosquito survival after fungal infection (p = 2.9 × 10^–8^, Cox HR = 1.6, 99% CI 1.3–2.0; Table 1 and Fig. 4A), whereas silencing of both isoforms simultaneously, by targeting the shared sequence present in the Rel2 messenger RNA did not influence mosquito survival outcome after fungal exposure (p = 0.395, Cox HR = 1.1, 99% CI 0.8–1.4; Table 1 and Fig. 4C). This result suggests the operation of a complex compensatory mechanism of Imd pathway signaling induced by the two Rel2 isoforms, such that the Imd/REL2-F axis of Imd signaling is protective against fungus-dependent mortality, while the simultaneous depletion of the short Rel2-S isoform reverses the Rel2-F antifungal activity. In control mosquitoes without fungus exposure, mosquito survival was not significantly influenced by treatment with dsRel2-F (p = 0.28, Cox HR = 1.1, 99% CI 0.9–1.4; Table1 and Fig. 4B) or dsRel2-S (p = 0.23, Cox HR = 1.1, 99% CI 0.9–1.4; Table 1 and Fig. 4B). Taken together, these results indicate that both the Toll and Imd immune pathways are involved in mosquito protection against fungus-dependent mortality. This is consistent with the above observation that downstream immune factors APL1C, LRIM1 and TEP1 controlled by these two pathways display anti-fungal activity, and suggests that other immune factors controlled by Toll and Imd also probably contribute to protection against fungal infection. Determining the identities of other putative protective factors will require further work.

### *A. coluzzii* displays sexually dimorphic mechanisms of immunity to fungal infection

**Figure 5:**
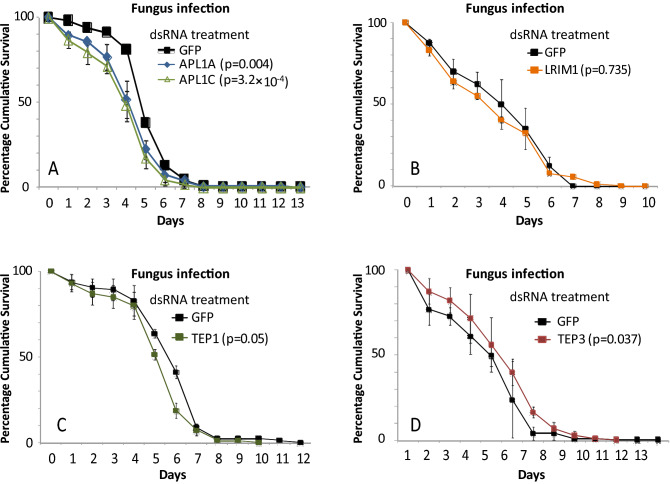
*Anopheles* antifungal immunity is sexually dimorphic. Graphs illustrate mortality curves of male mosquitoes after gene silencing followed by infection with *M. anisopliae*. (**A**) Silencing of APL1A and APL1C gene expression, (**B**) Silencing of LRIM1 gene expression, (**C**) Silencing of TEP1 gene expression and (**D**) Silencing of TEP3 gene expression. P-values obtained from Cox Regression are mentioned in brackets with the respective gene. Error bars represent the variability between three independent biological replicates. For each tested gene, 50 mosquitoes were tested in each of three biological replicates.

**Figure 6:**
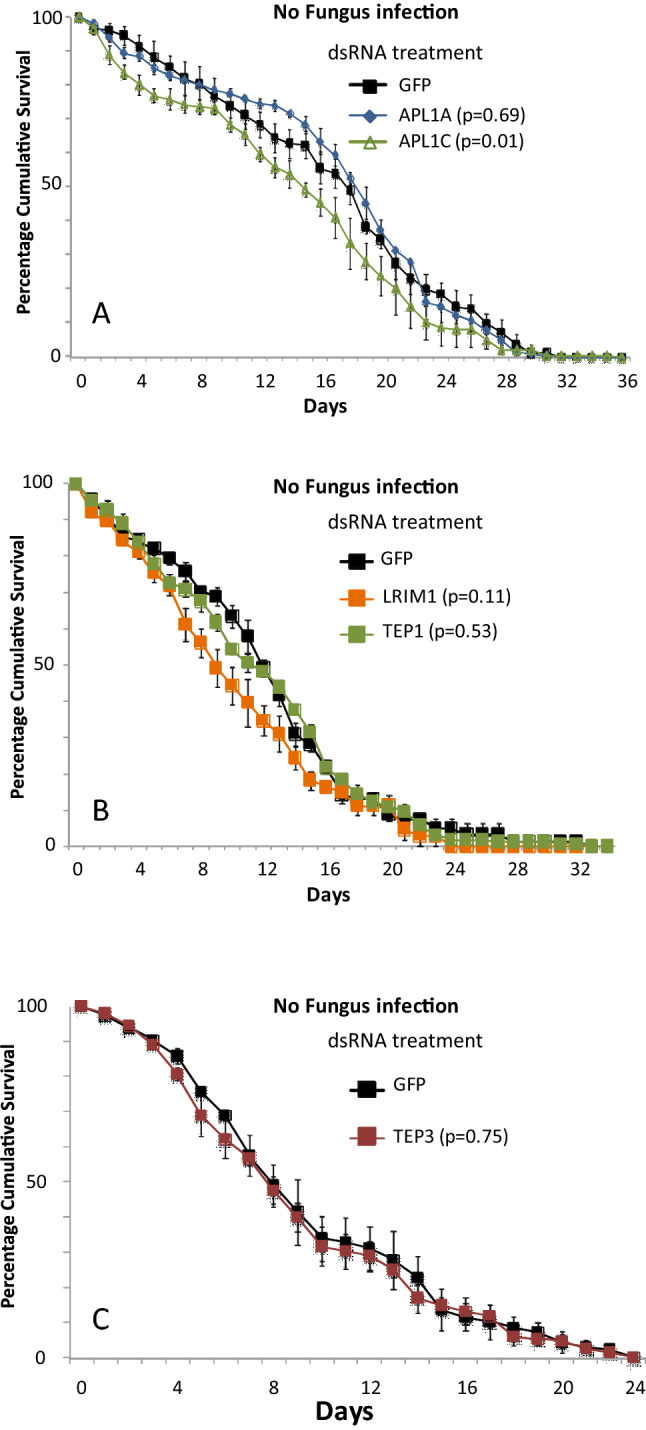
APL1C silencing in male *A. coluzzii* causes increased mortality The graphs illustrate mortality curves of male mosquitoes after gene silencing without infection (**A**) Silencing of APL1A and APL1C genes expression, (**B**) Silencing of LRIM1 and TEP1 gene expression, (**C**) Silencing of TEP3 gene expression. *p*-values obtained from Cox Regression are mentioned in brackets with the respective gene. Error bars represent the variability between three independent biological replicates. For each tested gene, 150 (50 per replicate) mosquitoes were used for all three biological replicates.

The biology and immunity of male mosquitoes is less studied than females because male mosquitoes do not bite and therefore have no role in disease transmission. Male *Anopheles* have shorter lifespans than females, likely due to their smaller body mass and consequent lower hydration levels ^27,28^. Males are less tolerant than females to environmental stressors such as fluctuation in temperature, humidity, and exposure to toxins such as *Bacillus thuringiensis var. israelensis* ^29–31^. Because fungal spores infect following contact with the insect cuticle, male mosquitoes are equally amenable as females to investigation of response to fungal infection. The function of the Mal2 candidate genes and functional partners, tested above in female mosquitoes, were here examined in males. Survival of male mosquitoes after fungal infection was significantly decreased by depletion of both APL1A and APL1C (APL1A, p = 0.004, Cox HR = 1.4, 99% CI 1.03–2.1; APL1C, p = 3.2 × 10^–4^, Cox HR = 1.6, 99% CI 1.13–2.1; Table 2 and Fig. 5A). However, distinct from female mosquitoes, there was no effect on male survival observed after depletion of LRIM1 or TEP1 (LRIM1, p = 0.73, Cox HR = 1.0, 99% CI 0.7–1.6, Table 2 and Fig. 5B; TEP1, p = 0.05, Cox HR = 1.3, 99% CI 0.9–1.9; Table 2 and Fig. 5C). In contrast, the complement paralog TEP 3 displayed a weak protective effect in males (p = 0.037, Cox HR = 0.8, 99% CI 0.6–1.06, p = 0.037; Table 2 and Fig. 5D). In control mosquitoes without fungus exposure, there was no effect on survival of male mosquitoes after treatment with dsAPL1A (p = 0.69, Cox HR = 1.0, 99% CI 0.7–1.2; Table 2 and Fig. 6A), or with dsLRIM1 (p = 0.11, Cox HR = 1.2, 99% CI 0.9–1.5; Table 2 and Fig. 6b), or with dsTep1 (p = 0.53, Cox HR = 0.9, 99% CI 0.7–1.2; Table 2 and Fig. 6B), or with dsTEP3 (p = 0.75, Cox HR = 1.0, 99% CI 0.8–1.4; Table 2 and Fig. 6C). Treatment with dsAPL1C may incur a slightly significant survival cost (p = 0.01, Cox HR = 1.3, 99% CI 0.9–1.7; Table 2 and Fig. 6A). These results indicate a previously unrecognized sexual dimorphism in the molecular mechanisms of immunity in male and female *A. coluzzii*. APL1C is required for protection from fungus infection in both males and females, while APL1A and TEP3 are protective in males but not in females. The differences are not directly due to sex chromosome dosage, because the LRR protein genes are located on chromosome 2, and the TEP family genes on chromosome 3. Further work will be required to understand the mechanism of sexually dimorphic immunity and its role in the natural biology of male mosquitoes.

**Table 2:** Hazard ratio HR (99%CI) and lethal median time (LT50) of adult male *A. coluzzii* mosquitoes after gene silencing by dsRNA treatment.

dsRNA	Fungus Infection					dsRNA	No Fungus Infection				
	HR	99% CI	*p*	LT50 ± S.E	n		HR	99% CI	*p*	LT50 ± S.E	n
APL1A	1.4	1.03–2.1	**0.004**	5 ± 0.1	187	APL1A	1.0	0.7–1.2	0.69	19 ± 0.5	200
APL1C	1.6	1.13–2.1	**3.2 × 10**^**–4**^	4 ± 0.1	153	APL1C	1.3	0.9–1.7	0.01	15 ± 1.0	187
LRIM1	1.0	0.7–1.6	0.735	4 ± 0.35	89	LRIM1	1.2	0.9–1.5	0.11	9 ± 0.6	166
TEP1	1.3	0.9–1.9	0.05	6 ± 0.13	98	TEP1	0.9	0.7–1.2	0.53	12 ± 0.9	157
TEP3	0.8	0.6–1.06	0.037	5 ± 0.27	132	TEP3	1.0	0.8–1.4	0.75	8 ± 0.5	157

*P*-values (*p*) were obtained from the Cox regression model. n corresponds to the total number of mosquitoes used for the Kaplan–Meier survival tests for each condition.

Significant values are in bold.

## Discussion

Here we show that mosquito genetics is a significant factor controlling differences in mosquito longevity following challenge with entomopathogenic fungus. Genetic mapping identified two loci influencing fungus-dependent mortality of mosquitoes. One of the mapped loci, Mal2, overlaps with a previously mapped locus influencing *Plasmodium falciparum* susceptibility in nature. Candidate gene exploration in the Mal2 locus led us to investigate the roles of Mal2 genes for LRR proteins, and their functional partners, in the mosquito response to entomopathogenic fungus. These genes are under the control of the Toll and Imd immune signaling pathways, and we showed that activity of both pathways is required for protection against *M. anisopliae* infection. The anti-*Plasmodium* factors APL1C, LRIM1, TEP1, and TEP3, all controlled by the Toll and Imd pathways, are required for protection of female mosquitoes against fungus-dependent mortality. In contrast to females, male *A. coluzzii* required the activity of APL1A, APL1C and TEP3 for protection against *M. anisopliae.* These results illustrate that the main mechanism described in *Anopheles* for immunity to *Plasmodium* infection, the LRR and complement system, in fact comprises a shared and fundamental protective mechanism against distinct eukaryotic pathogens, now extended also to an entomopathogenic fungus. This work supports and strengthens the concept of broad-spectrum innate immune protection in *Anopheles* mediated by combinatorial interactions among proteins encoded by different paralogs of at least the LRR and TEP families ^23,25^.

First described as an antibacterial factor promoting phagocytosis of both gram-negative and gram-positive bacteria ^32^, TEP1 was shown to protect against *Plasmodium* ^33^, as well as against the entomopathogenic fungus *B. bassiana* ^34^. The influence, if any, of LRR proteins was not reported in those studies. In the current work, we show that both LRIM1 and APL1C protect against *M. anisopliae* and we confirm the antifungal function of TEP1 in *A. coluzzii* females. Taken together, these data suggest that the LRR-TEP complex is required for broad spectrum protection against a range of pathogens.

While an influence of the enteric bacterial flora has been shown for *Anopheles* infection with *Plasmodium* ^35,36^, the effect of the mosquito microbiome on the antifungal response is poorly understood. Other studies performed on Aedes aegypti mosquitoes showed that the entomopathogenic fungus *B. bassiana* interacts with the gut microbiota to accelerate mosquito death ^37^, and induces a decrease of reactive oxygen species (ROS) activity that result in an increase of the gut microbiota ^38^. However, the endosymbiont *Wolbachia* in *Aedes* mosquitoes did not provide a protective advantage against entomopathogenic fungal infection ^39^. We previously showed that APL1 shapes the enteric bacterial consortium in *Anopheles* ^40^. Whether APL1-mediated antifungal response is microbiota-dependent is unknown and would require further work.

Formulations of fungal spores can be efficient as a mosquito biopesticide in field settings ^12,41^. In malaria endemic areas, co-infection events with *Plasmodium* are a likely scenario and studies have showed that co-infection with the fungus *B. bassiana* and rodent malaria, *Plasmodium chabaudi* displayed 80% reduction in transmission risk of malaria ^18^. However, a related study showed no effect of *B. bassiana* infection on *Anopheles* vector competence for two other *Plasmodium* species, *P. yoelli* and *P. falciparum* ^42^. The mosquito genetics and immune mechanisms were not dissected for *B. bassiana*, as they were in the current work for *M. anisopliae*.

The striking overlap of a genetic locus for protection from *M. anisopliae* with a locus for *Plasmodium* protection, as well as the shared identities of specific immune genes and signaling pathways against the two microbes, suggest that the investigation of co-infection with *M. anisopliae* and *Plasmodium* could be fruitful*.* An important outcome of entomopathogenic fungus infection is the strong reduction of the mosquito lifespan, and consequently of *Plasmodium* transmission efficiency. However, in addition, an expected result of the use of insecticides or other biocides, such as entomopathogenic fungi, in a target population is the selection of genetic mechanisms of resistance to the lethal agent. For chemical insecticides, the evolution of insecticide resistance is a purely negative outcome that must be managed to mitigate its harmful impact. Because the current study identifies key immune mechanisms that protect against both fungus and *Plasmodium*, this raises the possibility that selecting for genetic antifungal resistance by use of fungal biopesticide in the population might also augment the anti-*Plasmodium* resistance. Further work is needed to determine whether persistent exposure to fungal biopesticide over multiple mosquito generations can select for genetic resistance, and if so the phenotype of *Plasmodium* infection in such fungus-resistant mosquitoes.

In conclusion, the current study identified genetic loci, immune genes such as APL1C, LRIM1, TEP1, and TEP3 and their controlling signaling pathways that influence the outcome of fungal infection, and also influence susceptibility to *Plasmodium*. The similarity of immune mechanisms protecting against the two pathogens suggests that fungus-resistant mosquitoes could also display enhanced resistance to *Plasmodium*. Consequently, there could be an explicit operational advantage of selecting for fungus resistance in vector populations in order to promote naturally diminished malaria vector competence.

## Materials and methods

### Mosquitoes and fungi

Colonies of *Anopheles coluzzii* were each initiated with the eggs of single mated wild females to generate isofemale mapping lines (IMLs). The isofemale mothers of the IML pedigrees were collected in Mali (IML JBC03, Bancoumana village region) or Burkina Faso (IMLs 8, 13, 14, 27, 33, 34, Godin village region) using methods as previously described ^43^. The IMLs were used for the familiality study and for the genetic mapping. The Ngousso strain of *A. coluzzii* originates from mosquitoes collected in Yaounde, Cameroon ^44^. The Ngousso strain was used for the functional genomic study. Mosquitoes were reared under standard environmentally-controlled insectary conditions at 26 °C and 80% humidity, 12 h light/dark cycle with access to cotton soaked in 10% sucrose solution.

The experiments were carried out with *Metarhizium anisopliae* (ICIPE30 strain) spores, obtained from Dr. N.K. Maniania (International Centre of Insect Physiology and Ecology (ICIPE) germplasm centre, Nairobi, Kenya). This fungus strain was originally isolated from the stem borer, *Busseola fusca* in Kendu Bay, Western Kenya in 1999 and was passaged through *A. gambiae* to select for increased virulence to this host ^45^. The cultures were maintained on Sabouraud Dextrose Agar (SDA) at 25 ± 1 °C and 12:12 h L:D photoperiod. Conidia were collected by scraping two-week old sporulating cultures under aseptic conditions.

### *Anopheles* infection with fungal spores

For the familiality study and genetic mapping, in each replicate experiment, fifty 2–4 d old female mosquitoes were exposed to *M. anisopliae* strain ICIPE30 using described methods ^46^. Briefly, mosquitoes were exposed for 1 h to filter paper treated with spores formulated in ShellSol T at a surface coverage of 10e10 spores/m2. Control mosquitoes were identically exposed to papers treated with ShellSol T alone. After fungal exposure, the control and infected mosquitoes were maintained in the same conditions and mortality was monitored twice daily. Dead mosquitoes were removed and stored at −20 C before DNA extraction and genotyping.

For functional genomic experiments with candidate genes, in each experimental replicate 40–60 cold-anesthetized mosquitoes from the Ngousso colony, were placed on a petri dish containing one gram of *M. anisopliae* ICIPE30 spores and shaken gently for 30 s. dsRNA-mediated gene silencing followed by fungal challenge was replicated at least three times. Following fungal infection, mosquitoes were transferred to a holding cup with access to 10% sucrose solution. The holding cups were monitored daily, and the number of dead mosquitoes was recorded. The number of fungal spores attached to a mosquito was measured. This measurement was based on the number of spores collected from infected mosquitoes when vortexed in Tween 80 solution ^47^. The number of spores in the Tween 80 solution was counted with a Neubauer improved hemocytometer. The final calculation was based on three rounds of 5 mosquitoes each. Based on the hemocytometer count, each mosquito carried an average of 1.8 × 10^5^ spores/mosquito when infected with fungus by shaking on a petri dish with one gram of *M. anisopliae* spores for 30 s.

### Survival analysis

Kaplan–Meier survival tests were used to calculate the percent survival and median time to death for the pedigrees and significance of the observed differences was calculated using the non-parametric Log-Rank test. Cox regression was used to determine the differences in mosquito survival rates ^48^. A Cox regression model describes the increased or decreased likelihood of an event (in this case mosquito mortality), for each covariate, as a Hazard Ratio (HR). All experiments were tested for statistical differences within treatments across replicates. If no significant difference between individual replicate was detected, replicates were pooled. If differences between replicates were detected, replicates were included in the Cox regression model as a covariate to obtain an HR value adjusted for the difference observed across replicates. By either approach, the threshold for significance was defined as p < 0.01. All statistical analysis was performed using SPSS version 17.

### Genetic analysis

Age-matched mosquitoes were exposed to the same dose of *M. anisopliae* ICIPE30 as described above on day 0. Phenotypes were measured as deaths per day until all individuals in each pedigree were dead. Genetic mapping was carried out as described for *Plasmodium* infection ^15,19,49^. Individuals in pedigrees JBC03, IML8, IML13, IML14, IML27, IML33, IML34 were exposed to *M. anisopliae* ICIPE30, the quantitative phenotype was measured as days to death, and each individual was genotyped using the previously described 10 cM microsatellite map comprised of 30 microsatellite loci evenly spaced across the genome ^15^. To statistically test for significant linkage between genotype and time to death following infection with *M. anisopliae*. we compared the phenotype distribution for each genotype against the pooled phenotype distribution of all other genotypes. For every test, we computed the probability of encountering the observed difference under the null hypothesis of no linkage between this marker and mortality phenotype using the nonparametric Kolmogorov–Smirnov (KS) test as described ^15^. For each mapping pedigree, we performed approximately 60 allele tests and 100 genotype tests. An empirical permutation test was used to adjust for multiple testing, and adjusted p-values are reported.

### Gene silencing assays

Genes chosen as targets of double stranded RNA mediated gene silencing were selected based on documented anti-*Plasmodium* activity roles and roles in Toll, Imd pathways or a complementary pathway containing interacting leucine-rich repeat (LRR) and thioester-containing protein (TEP). Double-stranded (ds) RNAs were synthesized from PCR amplicons using the T7 Megascript Kit (Ambion) as described^22^. To silence the targeted gene, 500 ng of dsRNA was injected into the thorax of cold-anesthetized 1–2 d-old *A. coluzzii* adult using a nano-injector (Nanoject II, Drummond Scientific). For every experimental replicate, 60–70 mosquitoes were injected. dsGFP was used as control. Four days after the dsRNA injection 5 mosquitoes were randomly selected to verify the silencing of the target gene. The sequences of primers used for synthesis of dsRNA templates and for the verification of silencing efficiency are in supplementary Table S1.

## Supplementary Information

Supplementary Information.
