# Supplementary material for: Genetics and immunity of Anopheles response to the entomopathogenic fungus Metarhizium anisopliae overlap with immunity to Plasmodium
Source: Sci Rep. 2022 Apr 15;12:6315. doi: 10.1038/s41598-022-10190-3 (PMC9012835; doi:10.1038/s41598-022-10190-3)
Supplement: Supplementary file 1 — Supplementary Information. [file 41598_2022_10190_MOESM1_ESM.docx]

| **dsRNA** | **Primer Sequence** |
| --- | --- |
| T7-GFP-F | GAATTGTAATACGACTCACTATAGGGCATGGTGAGCAAGGGCGAG |
| T7-GFP-R | GAATTGTAATACGACTCACTATAGGGCTTACTTGTACAGCTCGTC |
| T7-APL1A-F | TAATACGACTCACTATAGGACTACCACCAGCCGAAAGATG |
| T7-APL1A-R | TAATACGACTCACTATAGGATCTGGTCTTGTATAGTACAATGG |
| T7-APL1B-F | TAATACGACTCACTATAGGACTCGCAAAGCTCAGCAAACAC |
| T7-APL1B-R | TAATACGACTCACTATAGGAGTGAGAACAAATAAGTTCAAAGTCC |
| T7-APL1C-F | TAATACGACTCACTATAGGAGGCCAAGAAGAACCGCAATCC |
| T7-APL1C-R | TAATACGACTCACTATAGGATCACAGTGATTTCAGGGTGTGC |
| S7’A | AGGCGATCATCATCTACGTGC |
| S7 B | GTAGCTGCTGCAAACTTCGG |
| APL1A-VF | GTAAACGAGCTGAGGACTGCGGTGCAGC |
| APL1A-VR | TCTGGTCTTGTATAGTACAATGGAACC |
| APL1B-VF | ACTCGCAAAGCTCAGCAAACAC |
| APL1B-VR | GTGAGAACAAATAAGTTCAAAGTCC |
| APL1C-VF | CTGCTGCAGGGGCTACACGCC |
| APL1C-VR | GGCCCAAGTAACATCATACAC |
| T7-Rel2-F | TAATACGACTCACTATAGGGCAACAGCAGCAACAACATC |
| T7-Rel2-R | TAATACGACTCACTATAGGGCACAGGCACACCTGATTGA |
| T7-Rel2F-F | TAATACGACTCACTATAGGAATCCGACGCAACGATACG |
| T7-Rel2F-R | TAATACGACTCACTATAGGGACCGCAATGTGAAGGATG |
| T7-Cactus-F | TAATACGACTCACTATAGGTGGTGCGTCGATTGCTGG |
| T7-Cactus-R | TAATACGACTCACTATAGGCTTTCGTTCAAGTTCTGTGC |
| T7-Tep1-F | TAATACGACTCACTATAGGTTTGTGGGCCTTAAAGCGCTG |
| T7-Tep1-R | TAATACGACTCACTATAGGACCACGTAACCGCTCGGTAAG |
| Tep1-VF | GGTGAATCAACGGTACGTTA |
| T7-Tep3-F | TAATACGACTCACTATAGGGCACCTCGACTGAGAAAGGTTTG |
| T7-Tep3-R | TAATACGACTCACTATAGGGCTGATTATTTATATAGTTTTAC |
| T7-Tep4-F | TAATACGACTCACTATAGGGCGGCGGAAAAGATCTCCCCG |
| T7-Tep4-R | TAATACGACTCACTATAGGGCGCGGCCGTCCGACAGCTGCG |
| Tep3-VF | ACCGCCAGGCGTACGTGATGG |
| Tep3-VR | CAAACCTTTCTCAGTCGAGGT |
| Tep4-VF | GGACCTCCATAATGCGGTGGC |
| Tep4-VR | CGGGGAGATCTTTTCCGCCAG |
